# Ethyl Caffeate Ameliorates Collagen-Induced Arthritis by Suppressing Th1 Immune Response

**DOI:** 10.1155/2017/7416792

**Published:** 2017-06-15

**Authors:** Shikui Xu, Aixue Zuo, Zengjun Guo, Chunping Wan

**Affiliations:** ^1^School of Pharmacy, Xi'an Jiaotong University, Xi'an 710061, China; ^2^Yunnan Institute for Food and Drug Control, Kunming 650011, China; ^3^School of Pharmacy, Yunnan University of Traditional Chinese Medicine, Kunming 650500, China; ^4^Central Laboratory, The No.1 Affiliated Hospital of Yunnan University of Traditional Chinese Medicine, Kunming 650021, China

## Abstract

The present study was designed to assess the antiarthritic potential of ECF in collagen-induced arthritis (CIA) and explore its underlying mechanism. *Methods.* In vitro, lymphocyte proliferation assay was measured by CCK-8 kit. In vivo, the therapeutic potential of ECF on CIA was investigated; surface marker, Treg cell, and intracellular cytokines (IL-17A and IFN-*γ*) were detected by flow cytometry. Th1 cell differentiation assay was performed, and mRNA expression in interferon-*γ*-related signaling was examined by q-PCR analysis. *Results.* In vitro, ECF markedly inhibited the proliferation of splenocytes in response to ConA and anti-CD3. In vivo, ECF treatment reduced the severity of CIA, inhibited IFN-*γ* and IL-6 secretion, and decreased the proportion of CD11b+Gr-1+ splenic neutrophil. Meanwhile, ECF treatment significantly inhibited the IFN-*γ* expression in CD4+T cell without obviously influencing the development of Th17 cells and T regulatory cells. In vitro, ECF suppressed the differentiation of naive CD4+ T cells into Th1. Furthermore, ECF intensely blocked the transcriptional expression in interferon-*γ*-related signaling, including IFN-*γ*, T-bet, STAT1, and STAT4. *Conclusion.* Our results indicated that ECF exerted antiarthritic potential in collagen-induced arthritis by suppressing Th1 immune response and interferon-*γ*-related signaling.

## 1. Introduction

*Elephantopusscaber* L. (*E. scaber*) belonging to the Compositae family is a small herb that is present in many parts of the world with mild and warm climate [1]. In folk medicinal practices, *E. scaber* have been used in many countries for the treatment of a number of diseases. In traditional Chinese medicine, *E. scaber*, popularly known as “Didancao,” is widely used as a diuretic antifebrile, antiviral, and antibacterial agent in treating hepatitis, rheumatic arthritis, bronchitis, cough associated with pneumonia, and arthralgia [2, 3]. In a previous study, we isolated 15 compounds from the ethanolic extract of this plant and screened them for immunological activities in ConA-induced murine splenocyte proliferation assay and found that ethyl caffeate (ECF), which was isolated from this plant for the first time, exhibited significant immunosuppressive activities [4]. Furthermore, it was determined that ECF markedly suppressed nitric oxide (NO) production and downregulated mRNA transcription of *i*NOS, L-1*β*, and IL-10 in LPS-treated RAW264.7 cells via suppressing the NF-*κ*B pathway, suggesting that ECF also possesses potential anti-inflammatory capabilities useful for treating various autoimmune diseases [5].

Rheumatoid arthritis (RA) is a chronic and systemic autoimmune disease, the main symptoms of which include pain and stiffness of joints and the progressive destruction of which may lead to disability [6]. RA is the most frequent of the chronic inflammatory joint diseases, with a prevalence of 0.5–1% in the industrialized world [7]. Although the etiological mechanisms of RA have not been fully elucidated, abundant evidence indicates that T cells are required for the initiation and chronicity of RA in both human and mouse models [8]. In light of the fact that there is an abundance of T cells in synovial tissues and fluids [9], recent studies showed that the imbalance of T cell subsets plays an important role in a variety of diseases, including RA [10]. Effector CD4^+^ T cell could be divided into Th1, Th2, Th17, and Treg subsets according to its differentiation and function [11, 12] Th1 cells secrete a large number of IFN-*γ*, which can promote cell-mediated immunity. Although the IFN-*γ* prevalence in pathogenesis of RA is controversial [13–15], IFN-*γ* was implicated as a major player in the pathogenesis of RA [16, 17].

Collagen-induced arthritis in DBA/1 mice is a well-established model of human RA [18–20]. The induction of severe chronic arthritis in naive mice requires type II collagen- (CII-) induced specific activation of T cells and B cells and is associated with Th1-polarized immune response. Moreover, IFN-*γ* derived from Th1 cell predominates during the induction and acute phases of this disease [21]. Two signaling pathways are known to be involved in IFN-*γ* production: IFN-*γ*/signal transducer and activator of transcription 1 (STAT1)/(T-bet)/IFN-*γ* signaling, and IL-12/STAT4/IFN-*γ* signaling [22].

To date, both the therapeutic potential of ECF in CIA and its mechanism of action remain unclear. This study aims to investigate the antiarthritic potential of ECF in CIA and explore the potential underlying mechanism of ECF for the treatment of RA. Our data demonstrate that ECF ameliorates collagen-induced arthritis by suppressing Th1 response and IFN-*γ* signaling pathway.

## 2. Materials and Methods

### 2.1. Experimental Animals

Female BALB/c and male DBA/1 mice (6–8 weeks old) were obtained from Shanghai Laboratory Animal Center of the Chinese Academy of Sciences and were housed in specific pathogen-free conditions (12 h light/12 h dark photoperiod, 22 ± 1°C, and 55 ± 5% relative humidity). All mice were allowed to acclimatize in our facility for 1 week before experiments. All experiments were carried out according to the institutional ethical guidelines on animal care and were approved by the Institute Animal Care and Usage Committee of the No. 1 Hospital Affiliated Yunnan University of Traditional Chinese Medicine.

### 2.2. Isolation of Ethyl Caffeate

*E. scaber* was purchased from Juhuachun medicine market of Yunnan province in 2010, and a voucher specimen (number DDC2010) was deposited at the herbarium of Yunnan University of TCM. Dried and powdered whole plants of *E. scaber* (10 kg) were extracted at room temperature with 100 L of 95% EtOH three times (100 L × 3). The extract was evaporated under reduced pressure to yield a dark brown residue (1.2 kg). The residue was then suspended in water and partitioned successively with 2.5 L of EtOAc to afford an EtOAc fraction (149 g). The EtOAc fraction was subjected to silica gel column chromatography (CC) eluted with CHCl_3_ and a gradient of CHCl_3_-MeOH to yield five fractions (Fr. 1–Fr. 5). Fraction 4 (7.9 g) was loaded to silica gel CC, eluting with CHCl_3_-acetone (9 : 1–7 : 3) in a gradient mode to yield four subfractions (Fr. 4a–Fr. 4d). Subfraction 4a was further separated with silica gel CC, eluted with CHCl_3_-acetone (8 : 2) to yield a residue, which was then recrystallized with MeOH to finally obtain the ethyl caffeate (210 mg).

### 2.3. Cell Preparation

Mice were sacrificed and spleens were removed aseptically. Splenocyte suspension was prepared as previously described [23] and resuspended in RPMI 1640 media containing 10% FBS and supplemented with penicillin (100 U/ml) and streptomycin (100 *μ*g/ml).

### 2.4. Proliferation Assay In Vitro

The proliferation of splenocytes or T cells in response to ConA, LPS, and anti-CD3/28 was measured by CCK-8 Kit. Briefly, BALB/c splenocyte suspension (5 × 10^5^ cells/well) was cultured with ConA (5 *μ*g/ml), LPS (10 *μ*g/ml), and anti-CD3 (5 *μ*g/ml; 145-2C11, BD Pharmingen) in the presence of ECF at indicated concentrations. The cultures were incubated for 48 h, 20 *μ*l of CCK-8 was then added to each well before the end of culture, and OD value was read at 450 nm. The MTT method was used to measure the cytotoxicity of the sample. Splenocytes (5 × 10^5^ cells/well) were cultured in triplicates in the absence or presence of ECF in a 96-well flat-bottomed plate (Costar) for 48 h. MTT (5 mg/ml) was pulsed for 4 h prior to the end of the culture. Upon removal of MTT/medium, 150 *μ*l of DMSO was added to each well, and the plate was agitated on an oscillator for 5 min to dissolve the precipitate. The assay plate was read at 570 nm using a microplate reader. All experiments were performed in triplicates independently.

### 2.5. Collagen-Induced Arthritis in DBA/1 Mice and Administration

Collagen was dissolved in 0.1 M acetic acid at 4°C overnight. Male DBA/1 mice were immunized at the tail base with 125 *μ*g of collagen emulsified in complete Freund's adjuvant (CFA) containing *Mycobacterium tuberculosis* strain H37Rv. Each mouse was then boosted with the same amount of collagen plus CFA 21 days later (taken as day 0). Starting from day 9 for 10 consecutive days, the mice were administered daily with ECF (50 mg/kg) or methotrexate (2 mg/kg) by p.o.

### 2.6. Evaluation of Severity of Arthritis

The severity of arthritis were assessed daily and expressed as a clinical score according to the following scale: 0 = normal; 1 = erythema or swelling of one or several digits; 2 = erythema and moderate swelling extending from the ankle to the midfoot (tarsal); 3 = erythema and severe swelling extending from the ankle to the metatarsal joints; 4 = complete erythema and swelling encompassing the ankle, foot, and digits, resulting in deformity and/or ankylosis. The scores of four limbs were summed up, and maximum score for each animal was therefore 16.

### 2.7. Splenocyte Proliferation Assay and Cytokines Production in CII-Immunized Mice

Splenocytes (5 × 10^5^ cells/well) were obtained from CII-immunized mice with or without ECF treatment and cultured in vitro with 100 *μ*g/ml CII stimulation. After 72 h, splenocyte proliferation assay was performed by CCK-8 Kit. Culture supernatants were harvested at 48 h to measure IFN-*γ*, TNF-*α*, IL-6, IL-17A, and IL-4 production by ELISA, in accordance with the manufacturer's instructions.

### 2.8. Flow Cytometry

Murine splenocytes were harvested and blocked with rat-anti-mouse CD16/CD32 (2.4G2, BD Pharmingen) and were then fluorescently labeled for 15 min at 4°C with the following mAbs diluted in PBS with 0.2% BSA: FITC-conjugated anti-mGr.1 (RB6-8C5, BD Pharmingen), PE-conjugated anti-mCD11b (M1/7), FITC-anti-mCD8a (53-6.7, BD Pharmingen), PE-conjugated anti-mB220 (RA3-682, BD Pharmingen), and PE-Cy7-conjugated anti-mCD4 (GK.1.5, BD Pharmingen).

Intracellular staining of cytokines was performed according to the method previously described [24, 25]. Briefly, splenocytes were restimulated for 4 h with PMA (50 ng/ml) and ionomycin (750 ng/ml) (Sigma-Aldrich) in the presence of Brefeldin A (Sigma-Aldrich). At the end of incubation, cells were collected and blocked with rat-anti-mouse CD16/CD32, then those were stained with PE-Cy7-anti-mCD4 antibodies (GK.1.5, Biolegend); cells were fixed, permeabilized, and stained intracellularly with fluorochrome-conjugated anti-mIL-17A (TC11-18H10.1, Biolegend) and anti-mIFN-*γ* (XMG1.2, Biolegend) using Foxp3 fixation/permeabilization reagents and protocols from eBioscience. Samples were acquired on a flow cytometer (FACSCanto™ II; BD Biosciences) and analyzed using FlowJo software (Tree Star).

### 2.9. Th1 Cell Differentiation

Purified CD4^+^ T cells were prepared using immunomagnetic negative selection (Miltenyi Biotec). Briefly, lymphocytes were allowed to react with CD4^+^ T cell biotin-antibody cocktail (biotin-conjugated monoclonal antibodies against CD8a, CD11b, CD11c, CD19, CD45R (B220), CD49b (DX5), CD105, anti-MHC class II, Ter-119, and TCR*γ*/*δ*) and then incubated with antibiotin microbeads, followed by magnetic separation. The purity of the resulting CD4^+^ T cell populations was examined using flow cytometry, and was consistently above 95%.

T-helper cell differentiation was conducted as previously described [26]. Briefly, naive CD4 T cells (0.4 × 10^6^) in 24-well plates (Costar) precoated with 5 *μ*g/ml of anti-CD3 (154-2C11, BioXcell) and 2 *μ*g/ml of anti-CD28 (37.51, BioXcell) were cultured in RPMI containing 10% FCS and polarizing cytokines at the various concentrations of ECF. The cytokines used were Th1, mIL-12 (10 ng/ml, Biolegend), and anti-mIL-4 (11B11, 2 *μ*g/ml, BioXcell). Cells stimulated in “neutral” conditions (anti-mIL-4 and anti-mIFN-*γ* without cytokines) were considered Th0 cells. The differentiated cells were harvested after 3 days and restimulated for 4 h with 50 ng/ml of PMA (Sigma-Aldrich) and 750 ng/ml of ionomycin (Sigma-Aldrich) in the presence or absence of Brefeldin A for flow cytometry.

### 2.10. TCR Engagement-Mediated T Lymphocytes Activation and qPCR Analysis

Purified CD4^+^ T cells were cultured in 24-well flat-bottom plates coated with 5 *μ*g/ml of anti-CD3 (145-2C11, BD Pharmingen) and 2 *μ*g/ml anti-CD28 (37.51, BD Pharmingen) in the absence or presence of ECF at indicated concentrations. Total RNA was isolated 16 h after stimulation with anti-CD3 and anti-CD28 using RNeasy Kit (Qiagen). 1 *μ*g of total RNA was used to synthesize cDNA using PrimeScript RT Master Mix Perfect Real Time (TaKaRa). IFN-*γ*, T-bet, STAT1, and STAT4 mRNA levels were detected by real-time quantitative PCR using SYBR Premix Ex Taq II kit (TaKaRa). Samples were assayed on Stratagene MX3000P Real-Time PCR machine (Agilent), and the relative expression level of the respective samples to *β*-actin was calculated with the delta-delta Ct. The gene-specific primers used were as follows:

IFN-*γ*: (sense) 5′-ATGAACGCTACACACTGCATC-3′

   (Antisense) 5′-CCATCCTTTTGCCAGTTCCTC-3′

T-bet: (sense) 5′-CCAGGAAGTTTCATTTGGGAAGC-3′

   (Antisense) 5′-ACGTGTTTAGAAGCACTG-3′

STAT1: (sense) 5′-TCACAGTGGTTCGAGCTTCAG-3′

     (Antisense) 5′-CGAGACATCATAGGCAGCGTG-3′

STAT4: (sense) 5′-GCAGCCAACATGCCTATCCA-3′

   (Antisense) 5′-TGGCAGACACTTTGTGTTCCA-3′

*β*-Actin: (sense) 5′-GGCTGTATTCCCCTCCATCG-3′

    (Antisense) 5′-CCAGTTGGTAACAATGCCATGT-3′.

### 2.11. Statistical Analysis

Data are expressed as mean ± s.e.m. of indicated experiments. Student's *t*-test was used to determine significance between two groups where appropriate. *P* < 0.05 was considered to be statistically significant.

## 3. Results

### 3.1. ECF Inhibits the Proliferation of Lymphocytes In Vitro

**Figure 1:**
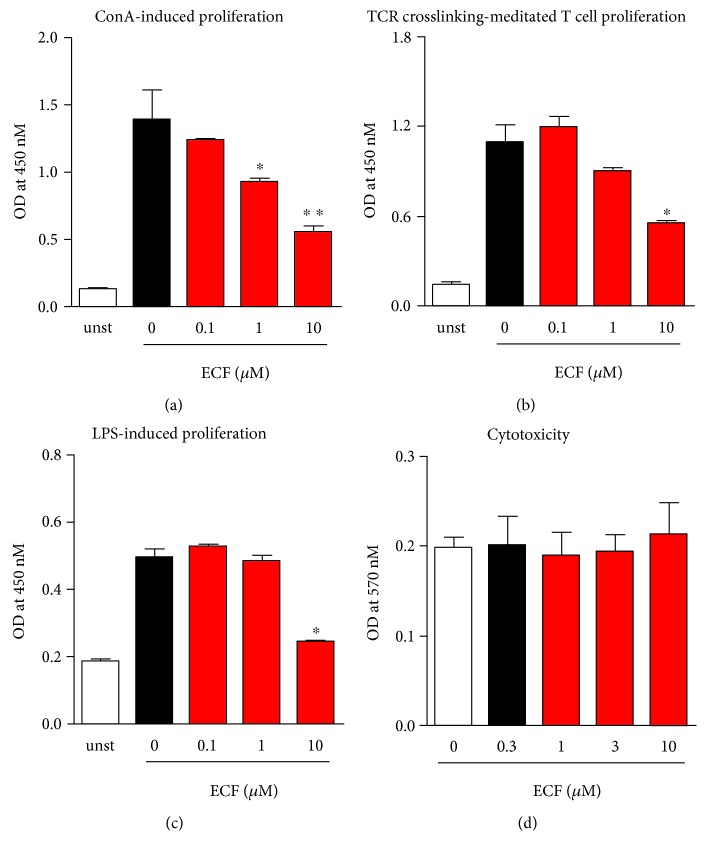
ECF inhibits lymphocyte proliferation in vitro. BALB/c splenocytes suspension (5 × 10^5^ cells/well) or purified T cells (2 × 10^5^ cells/well) were cultured with ConA (5 *μ*g/ml) (a), anti-CD3/CD28 (b), or LPS (10 *μ*g/ml) (c) in the presence of ECF at indicated concentrations for 48 h. The proliferation of lymphocytes was detected using CCK-8 Kit. The MTT method was used to measure the cytotoxicity of sample (d). Results presented are mean ± s.e.m., *n* = 3. ^∗^*P* < 0.05, ^∗∗^*P* < 0.01 versus control group.

To assess the immunosuppressive effect of ECF in vitro, lymphocyte proliferation assay was performed, in which in vitro stimulation of T cells with anti-CD3/28 serve to mimic the physiological crosslinking of TCR. ConA and LPS have been considered to be T and B cell mitogens, respectively. As shown in Figure 1, ECF significantly inhibits ConA-induced murine splenocyte proliferation (Figure 1(a)) as well as TCR crosslinking-mediated purified T cell proliferation (Figure 1(b)) in a concentration-dependent manner. ECF exerts immunosuppressive effects on LPS-induced B cell proliferation at the concentration of 10 *μ*M (Figure 1(c)). Importantly, ECF did not show obvious cell cytotoxicity on splenocytes at this concentration (Figure 1(d)). These data indicate that ECF can potentially serve as a promising therapeutic agent for treating autoimmune diseases.

### 3.2. ECF Attenuates Collagen-Induced Arthritis in DBA/1 Mice

**Figure 2:**
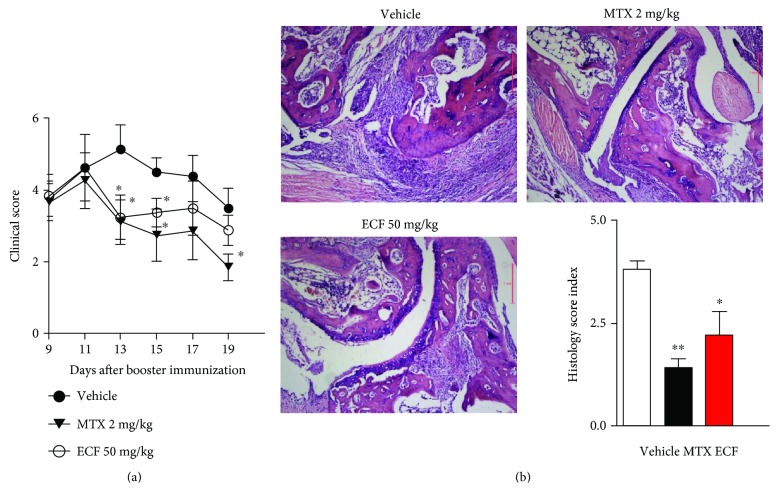
ECF attenuates collagen-induced arthritis in DBA/1 mice. Collagen-induced arthritis in DBA/1 mice was established via immunization twice with bovine collagen II in CFA. Mice were administered o.p. with vehicle, methotrexate (2 mg/kg), or ECF (50 mg/kg) once daily, starting from day 9 for 10 days. The severity of arthritis (a) and pathological changes to the joints (b) were evaluated using a clinical scoring system and H&E staining, respectively. Results presented are mean ± s.e.m., *n* = 7. ^∗^*P* < 0.05, ^∗∗^*P* < 0.01 versus vehicle group. IFN-*γ*, TNF-*α*, IL-6, IL-17A, and IL-4.

To investigate the antiarthritic potential of ECF, we adopted a well-established murine model of human rheumatoid arthritis, that is, collagen-induced arthritis in DBA/1 mice via immunization with bovine collagen II. The severity of the resulting arthritis in the presence or absence of ECF treatment was then evaluated using a clinical scoring system. We found that the clinical score for arthritis gradually increased in the vehicle group as the disease progressed and reached a peak at the day 13. In comparison, ECF treatment group exhibited significant reduction in clinical score from day 11 onwards, especially at the peak of arthritis progression (Figure 2(a)). The histopathological analysis of the joints by H&E staining indicated that the joints of CIA developed typical pathological changes, including severe cartilage and bone erosions, and infiltration of cells in the joints and synovial tissues and fluids. However, the number of infiltrated cells and the extent of bone destruction, as reflected by the histopathological score, show reduction in the ECF-treated group (Figure 2(b)). These data suggest that ECF possess therapeutic effects in treating arthritis.

### 3.3. ECF Inhibits Cytokine Production in CII-Specific Immune Responses

**Figure 3:**
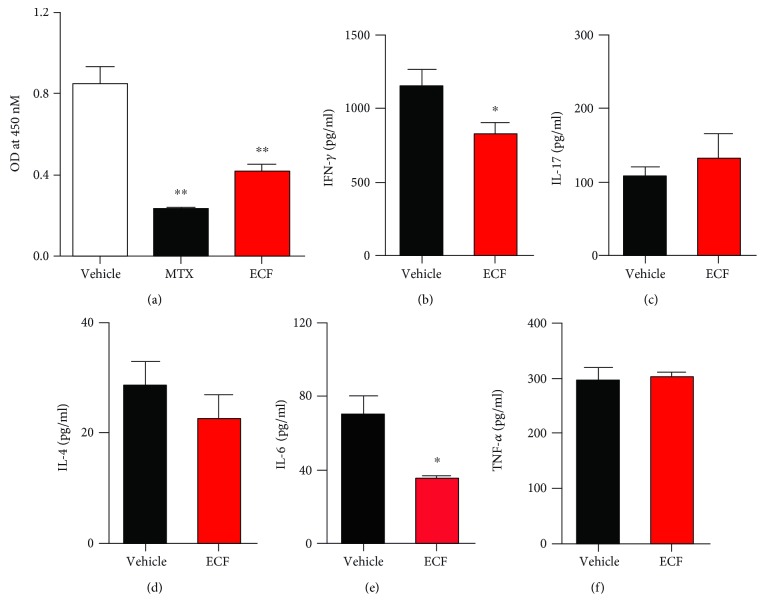
ECF inhibits CII-specific immune responses. Splenocytes (5 × 10^5^ cells/well) from CIA mice were recalled with 100 *μ*g/ml CII. After 72 h, splenocyte proliferation assay was performed using CCK-8 Kit. Culture supernatants were harvested at 48 h, and IFN-*γ*, TNF-*α*, IL-17A, and IL-4 levels were measured by ELISA. Results presented are mean ± s.e.m., *n* = 4. ^∗^*P* < 0.05, ^∗∗^*P* < 0.01 versus vehicle group.

To assess the effects of ECF on cell-mediated immune responses against CII, we investigated the secretion of cytokine in CII-specific immune responses. Splenocytes from CIA mice were recalled with CII for 48 h or 72 h to induce the production of cytokines and detected splenocyte proliferation, respectively. IFN-*γ*, IL-6, TNF-*α*, and IL-4 levels in the supernatants were determined by ELISA. As shown in Figure 3, ECF treatment significantly inhibited splenocyte proliferation (Figure 3(a)) and reduced the secretion of Th1-related cytokine (IFN-*γ*) in CII-induced specific immune response (Figure 3(b)). Since proinflammation cytokines such as IL-6 and TNF-*α* have a documented central role in the pathogenesis of arthritis, we further measured the levels of IL-6 and TNF-*α* under the same condition. ECF-treated CIA mice produced a lower level of IL-6 as compared to the vehicle group (Figure 3(d)), whereas no variation in TNF-*α* and IL-4 secretion levels were detected between them (Figures 3(c) and 3(e)). These data collectively indicated that treatment with ECF diminished the level of Th1 cytokine and some proinflammation cytokines.

### 3.4. ECF Treatment Significantly Reduces Accumulation of Neutrophils

**Figure 4:**
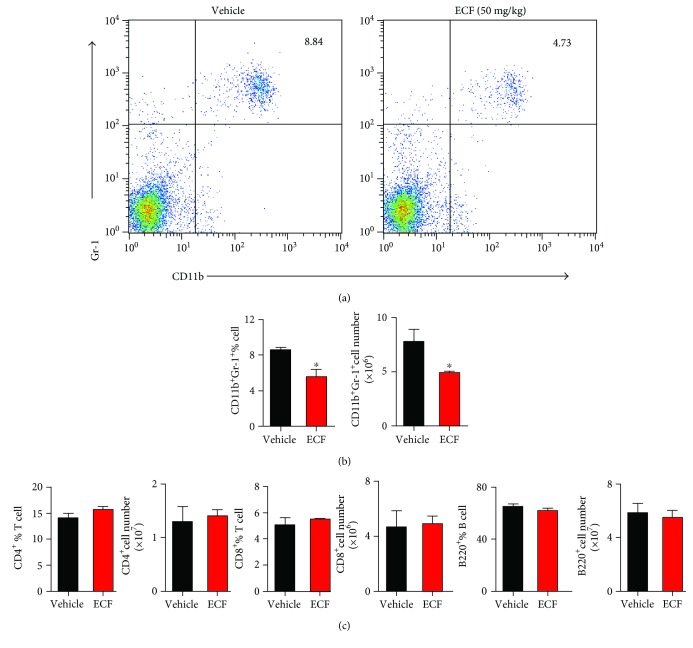
ECF treatment significantly reduces accumulation of neutrophils. Splenocytes from CIA mice were harvested and blocked with rat-anti-mouse CD16/CD32 and fluorescently labeled by incubation for 15 min at 4°C with the respective antibody. Samples were measured on flow cytometer and analyzed by FlowJo software. Results presented are mean ± s.e.m., *n* = 4. ^∗^*P* < 0.05, ^∗∗^*P* < 0.01 versus vehicle group.

Although the etiological mechanism of RA remains unclear, growing evidence indicates that T cells and B cells are involved in the pathogenesis of RA. Additionally, neutrophils are crucial for the pathogenesis of RA and other inflammatory conditions [27]. It has been reported that neutrophils in RA patients are highly activated in the circulation and synovial fluids and tissues [28]. In light of this, we analyzed the subset constitution of mouse spleens obtained from vehicle group mice and the ECF-treated group by flow cytometry (Figure 4()). Surface marker staining indicated that compared with the vehicle group, spleens from ECF-treated mice showed lower percentage of CD11b^+^Gr-1^+^ neutrophils but not CD4^+^, CD8^+^, or B220^+^ cells.

### 3.5. ECF Treatment Markedly Suppresses Th1 Cell Response in CIA

**Figure 5:**
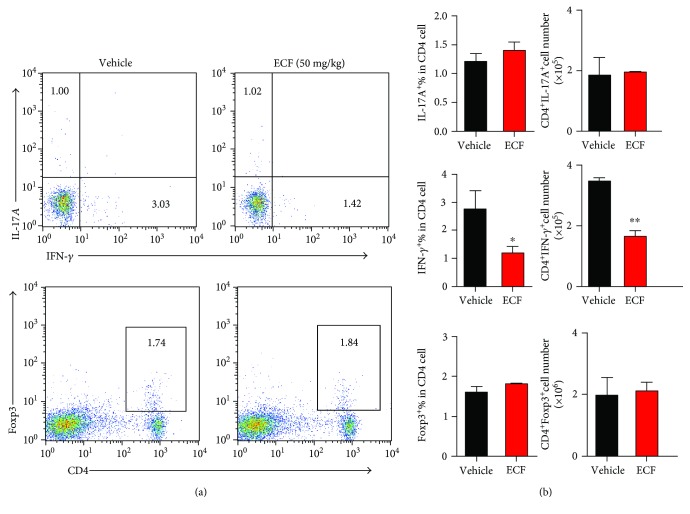
ECF treatment markedly suppresses Th1 cell response in CIA. Splenocytes from CIA mice were restimulated for 4 h with PMA and ionomycin in the presence of Brefeldin A. At the end of incubation, cells were collected and blocked with rat-anti-mouse CD16/CD32, and then those were stained with PE-Cy7-anti-mCD4 antibodies; cells were fixed, permeabilized, and stained intracellularly with fluorochrome-conjugated anti-mIL-17A, anti-mIFN-*γ*. Samples were acquired on flow cytometer and analyzed by FlowJo software. Results presented are mean ± s.e.m., *n* = 4. ^∗^*P* < 0.05, ^∗∗^*P* < 0.01 versus vehicle group.

T lymphocytes play a critical role in the pathogenesis of arthritis. Naive T cells could differentiate into pathogenic Th1 cells, proinflammatory T helper (Th17) cells, or tissue-protective induced T regulatory cells [29, 30]. To investigate whether ECF could regulate the development of Th1 and Th17 cells, we measured the intracellular cytokines expression in CD4^+^ T cells from CIA mice by flow cytometry (Figure 5). Compared to vehicle group, ECF treatment significantly inhibited IFN-*γ* expression in CD4^+^ T cells; the development of Th17 cells and T regulatory cells, however, was not obviously impacted.

### 3.6. ECF Inhibits Th1 Cell Differentiation

**Figure 6:**
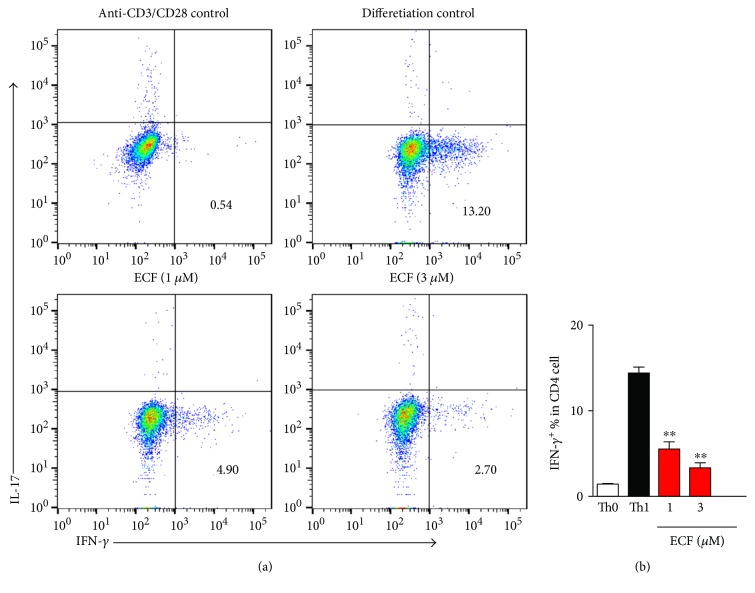
ECF inhibits Th1 cell differentiation. Naive CD4 T cells (4 × 10^5^) in 24-well plates precoated with anti-CD3 and anti-CD28 were cultured in RPMI containing 10% FCS and polarizing cytokines at various concentrations of ECF. The differentiated cells were harvested after 3 days and were restimulated for 4 h with PMA and ionomycin in the presence or absence of Brefeldin A for flow cytometry. Data were analyzed by FlowJo software.

In order to further investigate whether ECF could suppress Th1 differentiation, we stimulated murine naive T cells to induce Th1 differentiation in the presence of Th1 polarized condition (mIL-12 plus anti-mIL-4) and treated these cells with ECF. Strikingly, ECF impaired Th1 cell differentiation (Figure 6), in agreement with in vivo results, implying that the inhibitory effect of ECF on Th1 differentiation may contribute to its antiarthritic potential for treating RA.

### 3.7. ECF Suppresses IFN-*γ*-Related Pathway in TCR-Engagement-Mediated T Lymphocyte Activation

**Figure 7:**
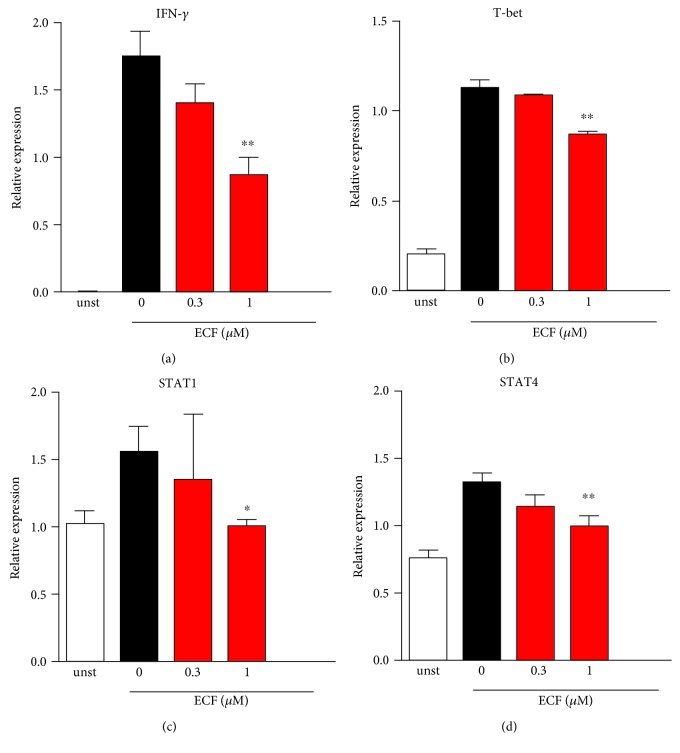
ECF suppresses IFN-*γ*-related pathway in TCR engagement-mediated T lymphocyte activation. Purified CD4^+^ T cells were stimulated with anti-CD3 (5 *μ*g/ml) and anti-CD28 (2 *μ*g/ml) for 16 h. Total RNA was isolated using RNeasy kits, and 1 *μ*g of total RNA was used to synthesis cDNA. Real-time quantitative PCR assay was carried out using SYBR Premix Ex Taq II kit, and relative quantification of mRNA expression was calculated as the fold increase using the delta-delta Ct; the housekeeping gene is *β*-actin. Results presented are mean ± s.e.m., *n* = 3. ^∗^*P* < 0.05, ^∗∗^*P* < 0.01 versus vehicle group.

Given that considerable evidence points to a critical pathogenic role of IFN-*γ* in both CIA model and patient, ECF significantly inhibits Th1 response and IFN-*γ* production in CIA model as described above. In order to further explore the mechanistic pathways of ECF on IFN-*γ* production, we measured the expression of IFN-*γ*-related signature genes such as IFN-*γ*, T-bet, STAT1, and STAT4 at the mRNA level upon TCR-engagement-mediated T lymphocyte activation (Figure 7). We found that transcriptional expression of IFN-*γ*, T-bet, STAT1, and STAT4 was significantly downregulated in the presence of various concentrations of ECF, suggesting that IFN-*γ*-related pathways are possibly involved in the immunosuppressive effect of ECF on T cell activation.

## 4. Discussions

It was reported that T lymphocytes play a pivotal role in the pathogenesis of cell-mediated autoimmune diseases and chronic inflammatory disorders [10]. Previous studies have indicated that ECF exhibits significant inhibitory effects on the proliferation of murine T lymphocytes induced by ConA, and the observed activities are not due to compound toxicity [4]. In vitro stimulation of T cells with anti-CD3/28 serves to mimic the physiological crosslinking of TCR. Importantly, ECF treatment markedly suppresses TCR-triggered T cell activation. The immunosuppressive activity of ECF on T cells revealed by the present study implies that ECF represents a potent therapeutic agent for the treatment of autoimmune diseases such as rheumatic arthritis. Moreover, collagen-induced arthritis (CIA) in DBA/1 mice is one of the many animal models used to study the pathogenic mechanisms of RA [31]. There is close resemblance in histopathology as well as in the production of inflammatory mediators such as chemokines, cytokines, proteases, and autoantibodies [13]. Surprisingly, oral administration of ECF ameliorates collagen-induced arthritis, including reduction in clinical score and infiltration of cells in the joints and synovial tissues and fluids. Furthermore, bone destruction and histopathological score are also reduced upon ECF treatment.

Depending on functions and cytokines produced, CD4^+^ T cells can be classified into four subsets, including T helper 1 (Th1), Th2, Th17, and CD4^+^ CD25^+^ T regulatory (Treg) cells. The imbalance of cytokine production by Th1/Th2/Th17 lymphocytes doubtlessly plays a crucial role in RA pathogenesis [10]. As Th1 cells mainly secrete interferon gamma (IFN-*γ*) and IL-2, Th1 cytokines are generally defined as cytokines that promote cell-mediated immune responses as well as activation of macrophage and neutrophils, leading to amplified production of proinflammatory cytokines [32]. Many studies have also provided evidence for the predominance of Th1 cytokine production in the synovial fluid from RA patients [33]. Intracellular cytokine staining reveals that ECF treatment inhibits the expression of IFN-*γ*-producing T cells compared to vehicle group when analyzed immediately ex vivo, whereas no difference was observed in Th17 cells or T regulatory cell response. This is consistent with the finding that IFN-*γ* production from T cells following restimulation with CII ex vivo was significantly reduced in ECF-treated mice. This response was antigen-specific and accompanied by reduced production of some inflammatory cytokines in the ECF-treated group, including reduction in the accumulation of neutrophils and the production of proinflammatory cytokines IL-6, which is mainly produced by activated macrophages and fibroblast-like synoviocytes (FLS). In RA, the level of IL-6 is also found to be elevated and correlates with radiological joint destruction. These results revealed that the inhibition of Th1-mediated immune response by ECF, especially the downregulation of IFN-*γ* expression, consequently limits the proinflammatory response and activation of neutrophils, which may contribute to the protective effect of ECF on CIA.

In adaptive immune response, IFN-*γ* is secreted by CD8^+^ T cells in control of infection, and by CD4^+^T helper1(Th1) subset, which promotes inflammatory response, clearance of intracellular pathogens, and class-switching to IgG 2a, IgG 2b, and IgG [34]. Differentiation of CD4^+^ T cells to the Th1 subset is driven primarily by IL-12 in the absence of IL-4 and TGF-*β*, which signal via factors such as signal transducer and activator of transcription 1 (STAT1), T-bet (also known as Tbx21), and STAT4 [35, 36]. Considerable evidence has indicated that pathogenic Th1-mediated immune response plays a crucial role in the pathogenesis of RA [33]. In vivo, ECF treatment markedly reduced the expression of IFN-*γ*-producing T cells compared to the vehicle group. Consistent with in vivo results, ECF also impaired Th1 cell differentiation and IFN-*γ* production in vitro, confirming that the inhibitory effect of ECF on Th1 differentiation is responsible for its antiarthritic potential.

The role of IFN-*γ* in the pathogenesis of RA is controversial, as both disease-limiting and disease-promoting activities of IFN-*γ* in the pathogenesis have been reported in the past decade [13]. Till now, there exists no satisfactory explanation for such dual effects in RA. It is reported that IFN-*γ*-deficient mice lead to disease exacerbation in CIA via IFN-mediated suppression of IL-17 [37]. Moreover, IFN-*γ* inhibits differentiation of monocyte/macrophage in osteoclasts through ubiquitin-proteasome-mediated degradation of TRAF6 [38]. Meanwhile, considerable evidence has revealed that IFN-*γ* may exacerbate RA: clinically, increased expression of IFN-*γ* has been detected in synovial tissues and fluids of RA patients [39–41], and for a small group of patients, intramuscular injection of anti-IFN antibodies appeared to be beneficial [42]. Administration of IFN-*γ* at the initial phase accelerated the onset and increased the incidence of arthritis [43, 44]. Furthermore, susceptible mice lacking IFN-*γ* of IFN-*γ* R exhibited decreased incidence and severity of CIA [45]. Many studies also suggested that the role of IFN-*γ* in RA may be associated with the disease stage and the use of CFA [46]. There are two signaling pathways involved in IFN-*γ* production: IFN-*γ*/signal transducer and activator of transcription 1 (STAT1)/(T-bet)/IFN-*γ*, signaling, and IL-12/STAT4/IFN-*γ* signaling [22]. PCR results indicated that ECF treatment downregulated the mRNA transcription of IFN-*γ*, STAT1, T-bet, and STAT4, providing molecular basis for its suppression of IFN-*γ* and the therapeutic effect of ECF in CIA.

Collectively, our results showed that ECF have antiarthritic effect in CIA mice, and its mechanism of action is tightly related to the blockade of IFN-*γ* signaling pathway. ECF may therefore present a promising agent for treating RA and other autoimmune diseases.
